# Boosting Smoking Cessation Intervention Utilization in Chinese Health Care Providers: A Randomized Controlled Trial of the “WeChat WeQuit” Medical Education Program

**DOI:** 10.1093/ntr/ntae166

**Published:** 2024-07-31

**Authors:** Yunfei Wang, Pu Peng, Zhenzhen Wu, Yuhang Liu, Chudong Wang, Jinsong Tang, Yanhui Liao

**Affiliations:** Department of Psychiatry, Sir Run Run Shaw Hospital, Zhejiang University School of Medicine, Hangzhou, Zhejiang, China; Department of Psychiatry, National Clinical Research Center for Mental Disorders, and National Center for Mental Disorders, Second Xiangya Hospital of Central South University, Changsha, Hunan, China; Department of Psychiatry, Sir Run Run Shaw Hospital, Zhejiang University School of Medicine, Hangzhou, Zhejiang, China; Department of Psychiatry, National Clinical Research Center for Mental Disorders, and National Center for Mental Disorders, Second Xiangya Hospital of Central South University, Changsha, Hunan, China; Department of Psychiatry, Ningbo Kangning Hospital, Ningbo, Zhejiang, China; School of Basic Medical Sciences, Central South University, Changsha, Hunan, China; School of Basic Medical Sciences, Central South University, Changsha, Hunan, China; Department of Psychiatry, Sir Run Run Shaw Hospital, Zhejiang University School of Medicine, Hangzhou, Zhejiang, China; Department of Psychiatry, Sir Run Run Shaw Hospital, Zhejiang University School of Medicine, Hangzhou, Zhejiang, China; Department of Psychiatry, National Clinical Research Center for Mental Disorders, and National Center for Mental Disorders, Second Xiangya Hospital of Central South University, Changsha, Hunan, China

## Abstract

**Introduction:**

In China, standard smoking cessation practices are rarely used by health care service providers (HSPs). WeChat, a popular social media app, has been widely used in China.

**Aims and Methods:**

In this single-blind, randomized trial, undertaken in China with 8-week interventions and follow-up to 34 weeks, 1887 HSPs were randomly selected to the intervention (*n* = 942) or control group (*n* = 945) from October 2020 to October 2021. The intervention group received regular smoking cessation training program messages from the professional team for 8 weeks and followed for 34 weeks. The control group received thanks messages for 8 weeks, and follow-up to 34 weeks. Both groups received a hard copy of the manual after randomization. The primary outcome measure was the utilization rate of behavioral and pharmacotherapy interventions for smoking patients from 9 to 34 weeks. This trial is registered at ClinicalTrials.gov (number NCT03556774).

**Results:**

HSPs in the intervention group demonstrated a better overall utilization rate of smoking cessation at 20-week follow-up compared to the control group (35.54% vs. 31.41%, *p* = .036). Additionally, both groups showed a significant increase in the adoption of various components of the 5A’s model—including “Assess,” “Assist: set a quit date,” “Assist: recommend cessation program,” “Assist: provide information,” “Assist: recommend medication,” and “Arrange”—at the 9-week follow-up relative to baseline. Notably, at the 20-week follow-up, the intervention group reported significantly enhanced utilization rates for all these components, except “Assist: set a quit date.”

**Conclusions:**

The “WeChat WeQuit” training program effectively enhanced smoking cessation intervention adoption among Chinese HSPs.

**Implications:**

“WeChat WeQuit” training program was effective in increasing the provision of effective tobacco cessation interventions by Chinese-speaking HSPs to patients with cigarette smoking, which could provide valuable insights into bridging the gap between need and services for smoking cessation in China.

## Introduction

With over 300 million smokers, cigarette smoking remains a major public health concern in China.^1^ In 2015, there were 933.1 million daily smokers in the world, and 6.4 million deaths (11.5% of global deaths) were attributable to cigarette smoking worldwide. Over three-quarters of deaths attributable to smoking were in men, and 52.2% took place in four countries (China, India, the United States, and Russia) with China having the highest proportion.^2^ Smoking cessation remains the single most effective prevention for lung cancer and other smoking-related health issues.^3^ However, long-term smoking cessation rates are very low (less than 10% quit rate).^4^ Our previous “Happy Quit” program in China (mobile phone-based text messaging interventions) showed only approximately 6% continuous abstinence rate at 24 weeks.^5,6^

In China, male physician smoking prevalence is high with few former smokers, and standard smoking cessation practices are rarely provided by HSPs.^7–9^ Evidence showed that smoking HSPs were less likely to ask about smoking status and advise smoking patients to quit, and physicians with higher perceived quality of their training in smoking cessation methods led to greater utilization of evidence-based cessation interventions.^10^ Thus, health care professionals should take more responsibility for providing smoking cessation services that are readily available, effective, and cheap. Taking the National Health Service (NHS) Stop-Smoking Service (SSS), a national evidence-based, effective, and incredibly cost-effective training program, for example, “Specialist” practitioners in the SSS reported higher success quit rates than “community” practitioners.^11^ Also clients who set a quit date with the SSS are more likely to maintain long-term abstinence.^12^ The SSS training program shows greater improvements in successful quit rates and provides a cost-effective way of increasing the number of people saved by the SSS.^13^ However, the availability of smoking cessation training programs in China is extremely limited, and the majority of cessation attempts end in relapse.^14^ Insufficient smoking cessation training programs for HSPs, and therefore insufficient smoking cessation services, would be the most important contributing factor to the low cessation rates reported in China.^7^ Furthermore, clinical studies on smoking cessation remain extremely inadequate in China.^15^ A sample from 21 cities in China reported that almost half of smokers intended to quit. However, the prevalence of smoking cessation among those urban-based smokers was only about 10%.^16^ There is an urgent need to improve the utilization of behavioral and pharmacotherapy interventions and to reach underserved populations in China.

Smoking cessation interventions delivered by digital media, such as text messages, WeChat, Facebook, e‐mails, web pages, and digital TV can be made widely available to those treatment-seeking smokers for little more than the cost of designing and testing the intervention. Previous trials on digital and social media interventions for smoking cessation have documented the long-term treatment effects.^17,18^ For example, the happy ending, with 12 months follow-up, demonstrated the efficacy of the fully automated digital multi‐media smoking cessation intervention.^18^ The txt2stop, a mobile phone text messaging smoking cessation program, also showed significantly improved smoking cessation rates at 6 months.^19^

Since its first release in 2011, WeChat (Chinese: 微信; pinyin: Wēixìn; literally: “micro-message”) has become the most popular social media app in China (https://en.wikipedia.org/wiki/WeChat). It has been widely used, either by individuals or groups, among HSPs and patients to promote human health. With the rapid increase of WeChat users during the past several years, most of health care settings have built WeChat public service platforms to provide instant medical service.^20^ There is evidence to show that WeChat-based medical services can improve the quality of care and treatment efficacy. Take smoking cessation services, for example. A sample of 88 patients with coronary heart disease who underwent percutaneous coronary intervention were randomly selected to WeChat Groups with smoking cessation intervention or without intervention. This showed that using the WeChat Group-based smoking cessation improved these patients’ quit rates.^21^ Two similar smoking cessation studies in China showed that combination therapy of varenicline with a WeChat-based service platform is better than varenicline alone for patients with chronic obstructive pulmonary disease.^22,23^

In order to minimize the huge gap between the shortage and demand for smoking cessation training programs and smoking cessation services, we proposed this “WeChat WeQuit” smoking cessation training program for Chinese-speaking HSPs to increase the utilization of behavioral and pharmacotherapy interventions for cigarette smoking cessation.

## Materials and Methods

This study was a Registered Report. The approved stage 1 protocol can be found at https://doi.org/10.17605/OSF.IO/8TQAU. The data, anonymized study data, digital materials, code, and the laboratory log of this study can be found at https://osf.io/4h35q/.

### Study Design, Sample, and Recruitments

This was a WeChat-based, single-blind, two-arm RCT undertaken in China without regional restrictions between June 2018 to May 2022. Eligible Chinese-speaking HSPs were randomly selected for the intervention or control groups in a 1:1 ratio(by randomizeR, https://CRAN.R-project.org/package=randomizeR). The inclusion criteria included: (1) Chinese-speaking HSPs; (2) Know how to use WeChat; (3) Use WeChat on a daily basis; and (4) Willing to provide informed consent to participate in the study. Non-Chinese speakers, non-HSP, and those who do not use WeChat or fail to provide informed consent were excluded.

We advertised this service in hospitals, private clinics, pharmacies and online (eg WeChat, websites, such as http://www.dxy.cn/, QQ). Potential participants would register their interest by sending WeChat messages, text messages or making a call. Research assistants then contacted respondents to assess eligibility and collect baseline data. A research assistant assigned participants to either an intervention or control group. Participants, investigators and other research personnel were masked to treatment allocation. This trial was carried out in two phases, including the pilot study and the main study. The methods and results of the pilot study were detailed in the Supplementary Materials.

### Study Procedure

**Figure 1.**
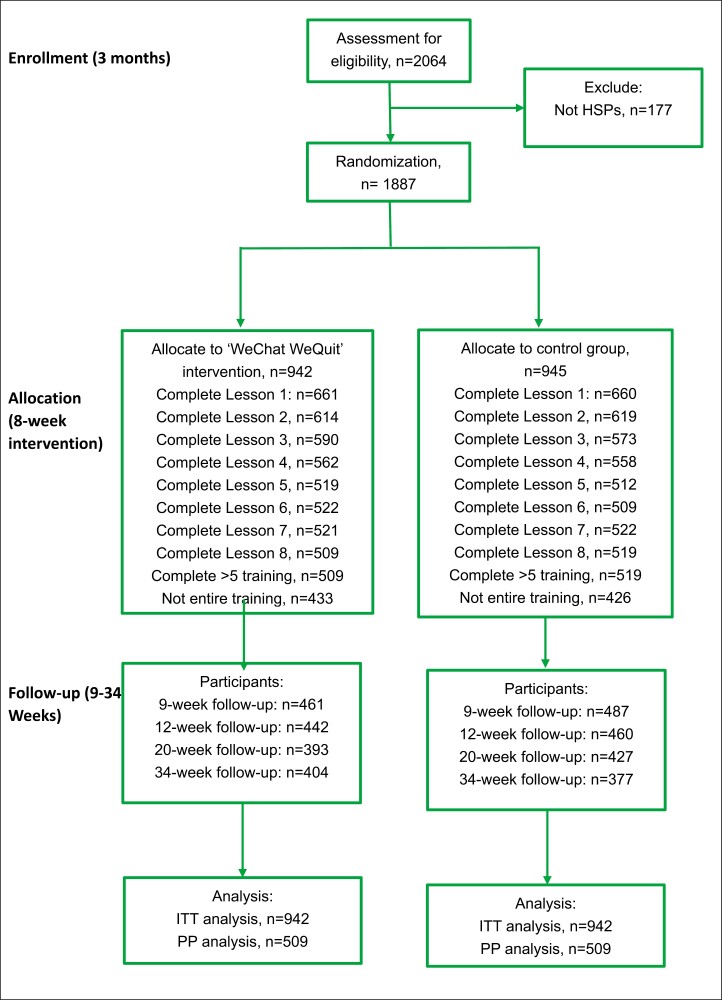
Flowchart for the main study.

The flowchart for the main study is shown in Figure 1. This trial recruited about 2000 HSPs in the main study with a 26-week follow-up. They could submit any comments or suggestions on the project at the end of the project.

#### Intervention Group

Participants who were allocated to the intervention group received regular smoking cessation training program messages (WeChat group messages and one-to-one individual messages, mainly including text, picture, and video messages) by a professional team. One to six messages were sent per week for 8 weeks. One to six messages were sent per month until the end of the 26-week follow-up.

#### Control Group

Control group participants did not receive any smoking cessation messages from the professional team. They received messages thanking them for being in the study and reminding them of the time until the 26-week follow-up. One to six messages were sent per week for 8 weeks. One message was sent per month until the end of the 26-week follow-up.

#### For Both Groups

A hard copy of the behavioral and pharmacotherapy interventions manual was sent to each HSP by mail after randomization. All-HSPs received WeChat-based questions at the first 8 weeks and at the follow-up 9, 12, and 20 weeks until 34 weeks about their smoking cessation services and patients’ 7-day point prevalence of abstinence and continuous smoking abstinence (details see Appendix 5). Knowledge about behavioral and pharmacotherapy interventions for smoking cessation before and after 8 weeks of training.

#### Outcomes of the Main Study

The primary outcome was the utilization rate (ie rate = smokers treated divided by smokers seen) of behavioral and pharmacotherapy interventions by HSPs for smoking patients from 9 to 34 weeks. The number of smokers seen by each HSP, as well as the number of smokers given treatment by each HSP were measured. The utilization rate will be recorded by each HSP. The secondary outcome measure was the proportion of smoking patients with continuously self-reported abstinence at week 9, 12, 20, and 34 follow-ups. The association between particular behavioral change techniques or medications by HSPs and higher rates of successful quitting will be measured as well. A third outcome would explore the potential wider influence of the “WeChat WeQuit” training program in China.

### Measurements

We collected baseline data on gender, age, education levels, clinical sites, years of practice, types of practice, and cigarette smoking characteristics for smoking providers only as covariates. Program acceptability was measured by questions for assessing program acceptability in Supplementary Appendix 3. Knowledge about behavioral and pharmacotherapy interventions for smoking cessation before and after 8 weeks was measured on a 100-point scale in Supplementary Appendix 4. The utilization rate of interventions for smoking patients from 9 to 34 weeks was measured by items from Supplementary Appendix 5. The self-reported continuous smoking abstinence defined as no more than five cigarettes smoked in the past week at 4 weeks follow-up and no more than five cigarettes smoked since the start of the abstinence period at 6 months of follow-up was also collected.

### Sample Size and Power Calculation

The sample size assessment and power calculations are mainly based on the primary outcome of “utilization rate of behavioral and pharmacotherapy interventions for cigarette smoking patients from week 8 to week 34,” as well as the secondary outcome measure of “quit rates.” On the basis of the results of the English National Health Service’s (NHS) Stop-Smoking Services research paper^11^ with a total of 573 specialist practitioners and 466 community practitioners, we estimated that, for assessing the utilization of counseling, 124 HSPs (62 HSPs in each group) are required to have an 80% chance of detecting, as significant at the 5% level, an increase in the primary outcome measure from 58.1% in the control group to 80.7% in the intervention group, for assessing the utilization of advising on stop-smoking medications, 348 HSPs are required to have an 80% chance of detecting as significant, at the 5% level, an increase in the primary outcome measure from 85.9% in the control group to 94.7% in the intervention group. For assessing 4-week biologically verified quit rates, 434 HSPs are required to have an 80% chance of detecting as significant, at the 5% level, an increase in the primary outcome measure from 50.4% in the control group to 63.6% in the intervention group. However, a larger sample size will be required to assess 24-week continuous quit rates between the two groups.

We then assessed the sample size based on previous RCTs of smoking cessation programs and Internet interventions, which suggest that quit rates for smokers may be as high as 10%–20% in the treatment group and as low as 5% in the control group.^24–26^ A conservative estimate of 10% (treatment) and 5% (control) self-reported prevalence abstinence at 12- and 24-week follow-up was made. A sample size of 864 will provide a power level of 0.80, and a type-I error rate of 0.05 to detect these cessation rates in the most conservative outcome of the study self-reported prevalence abstinence. However, considering relatively large dropoffs,^27^ a total sample size of 2200 participants in this study (1100 in each arm) will have more than a 95% chance of detecting a significant difference.

### Data Analysis

Statistical analyses were conducted with R software (https://www.r-project.org/) and SPSS version 22 (IBM Corp. Released 2013. IBM SPSS Statistics for Windows, Version 22.0. Armonk, New York: IBM Corp.). Data were double-checked, and groups were blinded to statisticians. Basic information (including demographic characteristics and cigarette smoking characteristics) at baseline and the overall quality and efficacy of the “WeChat WeQuit” training program (including program acceptability, HSPs’ knowledge and competencies, and utilization rate) between study groups using the χ^2^ (chi-square) or Fisher exact test for categorical data and the *t* test or Wilcoxon rank-sum test for continuous data were collected. Smoking cessation outcomes will be recorded by HSPs. The utilization rate of interventions will be compared between HSPs in the intervention group and the control group using a mixed-effects statistical model. As for smoking status and quit rates, all-smoking patients who visited HSPs from this trial will be recorded and analyzed with an “intention-to-treat” analysis. Self-reported continuous abstinence from 1 week to 24 weeks after the quit date was compared between two groups using the χ^2^. The independent variables were intervention versus the control condition, assessment points, and covariates identified in the preliminary analysis. This model was estimated using maximum likelihood estimation. All-tests will be 2-tailed. A two-sided *p* < .05 will be used to determine statistical significance.

## Result

### Sample Characteristics

Between October 2020 and October 2021, a total of 2064 individuals were assessed for eligibility criteria (Figure 1). Subsequently, 1887 HSPs were randomized into two groups: 942 allocated to the WeChat intervention group and 945 to the control group. Baseline demographic characteristics were well-matched across both cohorts, as summarized in Table 1. The participants included 787 males (41.7%) and 1100 females (58.3%), with a mean age of 37.02 years and a mean working duration of 13.48 years. Predominantly, participants were of Han ethnicity (*n* = 1767; 93.6%) and possessed a bachelor’s degree or below (*n* = 1381; 73%). The majority were employed in tertiary care hospitals (*n* = 1134; 60.1%), while secondary hospitals employed 524 (27.7%), and the remaining 229 (12.1%) were affiliated with primary care facilities or other health services. Professional titles were distributed as follows: junior (*n* = 645; 34.2%), middle (*n* = 800; 42.4%), and senior (*n* = 442; 23.4%). Approximately one-tenth and two-fifths of the participants worked in the Department of Respiratory Medicine or the Department of Mental Health. Current smokers accounted for 5.5% (*n* = 104) of the HSPs.

**Table 1.** Baseline Characteristics of the Participants

Variable	Overall, *N* = 1887^a^	Control group, *N* = 945^a^	“WeChat WeQuit” intervention group, *N* = 942^a^	*p*-Value^b^
**Gender**				.86
Male	787 (42%)	396 (42%)	391 (42%)	
Female	1100 (58%)	549 (58%)	551 (58%)	
**Age, y**	37.02 (8.13)	36.88 (8.15)	37.17 (8.11)	.44
**Nationality**				.56
Han	1767 (94%)	888 (94%)	879 (93%)	
Non-Han	120 (6.4%)	57 (6.0%)	63 (6.7%)	
**Education level**				.97
Bachelor’s degree or below	1381 (73%)	692 (73%)	689 (73%)	
Masteral or doctoral degree	506 (27%)	253 (27%)	253 (27%)	
**Working duration, y**	13.48 (9.06)	13.29 (9.14)	13.67 (8.98)	.37
**Hospital level**				.31
Tertiary hospital	1134 (60%)	565 (60%)	569 (60%)	
Secondary hospital	524 (28%)	274 (29%)	250 (27%)	
Primary hospital	229 (12%)	106 (11%)	123 (13%)	
**Hospital nature**				.28
Public hospital	1730 (92%)	876 (93%)	854 (91%)	
Private hospital	134 (7.1%)	59 (6.2%)	75 (8.0%)	
Clinic	23 (1.2%)	10 (1.1%)	13 (1.4%)	
**Major**				.5
Psychiatry or mental health	783 (41.49%)	384 (40.63%)	399 (42.36%)	
Respiratory medicine	251 (13.30%)	121 (12.80%)	130 (13.80%)	
Others	853 (45.20%)	440 (46.56%)	413 (43.84%)	
**Professional title**				.35
Junior	645 (34%)	321 (34%)	324 (34%)	
Middle	800 (42%)	414 (44%)	386 (41%)	
Senior	442 (23%)	210 (22%)	232 (25%)	
**Smoking status**				.49
Nonsmokers	1514 (80%)	749 (79%)	765 (81%)	
Ex-smoker	269 (14%)	139 (15%)	130 (14%)	
Current smokers	104 (6%)	57 (6%)	47 (5%)	
**Utilization rate of standard 5A’s intervention,%**				
Ask	62.87 (36.06)	63.85 (35.67)	61.88 (36.44)	.24
Advice	64.84 (35.60)	65.22 (35.68)	64.47 (35.54)	.65
Assess	41.05 (33.59)	40.27 (33.51)	41.82 (33.67)	.32
Assist: set a quit date	27.83 (32.74)	27.37 (32.65)	28.29 (32.83)	.54
Assist: recommend cessation program	32.51 (34.59)	32.12 (34.75)	32.89 (34.44)	.63
Assist: provide information	37.24 (34.56)	36.98 (34.38)	37.50 (34.76)	.74
Assist: recommend medication	28.83 (32.34)	28.11 (31.98)	29.56 (32.70)	.33
Arrange	27.86 (32.76)	27.27 (32.49)	28.44 (33.04)	.44
**Competence of training**				
Lesson 1	1313 (70%)	655 (69%)	658 (70%)	.80
Lesson 2	1228 (65%)	616 (65%)	612 (65%)	.92
Lesson 3	1158 (61%)	568 (60%)	590 (63%)	.28
Lesson 4	1129 (60%)	556 (59%)	573 (61%)	.40
Lesson 5	1094 (58%)	541 (57%)	553 (59%)	.54
Lesson 6	1108 (59%)	540 (57%)	568 (60%)	.18
Lesson 7	1092 (58%)	539 (57%)	553 (59%)	.48
Lesson 8	1028 (54%)	519 (55%)	509 (54%)	.71
Finish the training	1028 (54%)	519 (55%)	509 (54%)	.70
**Follow-up**				
8-wk follow-up	948 (50%)	487 (52%)	461 (49%)	.27
10-wk follow-up	902 (48%)	460 (49%)	442 (47%)	.46
20-wk follow-up	820 (43%)	427 (45%)	393 (42%)	.13
34-wk follow-up	781 (41%)	377 (40%)	404 (43%)	.19

^a^Mean (SD); *n* (%).

^b^Wilcoxon rank sum test; Pearson’s chi-squared test.

At baseline, the utilization rates of the “Ask” and “Advice” components of the 5A’s intervention were high (62% and 64%, respectively). Conversely, the employment of the remaining 5A’s strategies was considerably lower, with reported utilization rates ranging from 27% to 41%. No significant differences in the utilization rates of the 5A’s intervention were observed between the two groups at baseline.

### Completion Rate of Intervention and Follow-Up

Of the individuals in the WeChat intervention cohort, 54% (*n* = 509) completed the 8-week training (defined by finishing the study of at least 5 out of 8 lessons), paralleled by a 55% completion rate (*n* = 519) in the control group. There was no significant difference in the completion rate of any lessons between the two groups (all *p* > .05).

Follow-up participation rates in the “WeChat WeQuit” intervention group were 49%, 47%, 42%, and 43% at 8, 12, 20, and 34 weeks, respectively. Correspondingly, in the control group, follow-up rates were 52%, 49%, 45%, and 40%. The follow-rates did not differ between the two groups, with 60% of the intervention group and 59% of the control group participating in at least one follow-up session.

### Effectiveness of WeChat Intervention in the Main Study

Table 2 presented the study outcomes. Regarding the primary outcome, the overall utilization rate at the 20-week follow-up was significantly higher in the intervention group compared to controls (35.54% vs. 31.41%, *p* = .036). No significant differences were observed at 8, 12, and 34-week follow-ups.

**Table 2:** ITT Analysis of Outcomes for All-Participants in the WeChat Intervention and Control Groups (*n* = 1887)

Outcome	WeChat intervention group, *n* = 942	Control group, *n* = 945	Adjusted mean difference (standard error)	*p*-value^a^
***Primary outcome***				
**Overall utilization rate, %**				
8-wk follow-up	41.52 (33.52)	42.75 (34.36)	–0.39 (2.06)	.69
12-wk follow-up	32.05 (33.69)	31.26 (35.14)	1.88 (2.14)	.38
** 20-wk follow-up**	**35.54 (34.61)**	**31.41 (34.78)**	**4.63(2.21)**	**.036**
34-wk follow-up	35.27 (33.97)	34.14 (33.46)	2.24 (2.26)	.32
*** Secondary outcome***				
** Self-reported continuous abstinence rate of patients treated by HSPs, %**				
8-wk follow-up	4.88 (12.44)	6.80 (24.96)	–0.63 (1.64)	.69
12-wk follow-up	21.52 (28.22)	21.58 (29.26)	1.13 (1.77)	.52
20-wk follow-up	21.44 (28.13)	19.17 (26.51)	2.74 (1.83)	.14
34-wk follow-up	19.63 (27.89)	18.80 (26.75)	2.03 (1.87)	.27
** Self-reported initiation rates of smoking cessation among patients treated by HSPs, %**				
8-wk follow-up	32.01 (28.76)	33.86 (30.47)	–0.55 (1.81)	.76
12-wk follow-up	25.07 (28.96)	24.73 (30.31)	2.16 (1.88)	.25
20-wk follow-up	27.92 (29.38)	25.87 (30.20)	3.43 (1.94)	.08
34-wk follow-up	28.63 (30.43)	27.50 (29.31)	2.69 (1.98)	.18
*** Other outcomes***				
** Utilization rate of 5A’s intervention, %**				
** Ask**				
8-wk follow-up	65.87 (34.09)	64.48 (36.33)	4.34 (2.41)	.07
** 12-wk follow-up**	51.42 (40.56)	47.47 (42.48)	**6.59 (2.45)**	**.007**
** 20-wk follow-up**	56.63 (40.03)	49.87 (42.23)	**9.56 (2.54)**	**<.001**
34-wk follow-up	53.45 (40.93)	52.89 (42.31)	3.22 (2.58)	.21
** Advice**				
** 8-wk follow-up**	69.62 (33.92)	65.43 (37.48)	**5.16 (2.43)**	**.034**
** 12-wk follow-up**	53.97 (41.93)	49.19 (43.12)	**5.82 (2.48)**	**.018**
** 20-wk follow-up**	58.45 (41.32)	51.57 (43.29)	**7.91 (2.57)**	**.002**
34-wk follow-up	56.67 (41.77)	56.72 (42.81)	1.62 (2.61)	.53
** Assess**				
8-wk follow-up	52.53 (31.59)	49.70 (34.82)	2.53 (2.18)	.24
12-wk follow-up	41.51 (36.24)	38.31 (37.10)	2.81 (2.22)	.20
** 20-wk follow-up**	47.86 (37.71)	39.22 (37.46)	**7.54 (2.30)**	**.001**
34-wk follow-up	45.15 (37.28)	42.10 (37.11)	2.54 (2.34)	.28
** Assist: set a quit date**				
8-wk follow-up	38.63 (33.12)	38.71 (34.75)	-0.69 (2.06)	.74
12-wk follow-up	31.24 (34.01)	28.56 (33.82)	1.62 (2.10)	.44
20-wk follow-up	34.09 (34.75)	29.77 (33.25)	2.78 (2.18)	.20
34-wk follow-up	33.43 (34.23)	31.91 (33.42)	0.58 (2.21)	.79
** Assist: recommend cessation program**				
8-wk follow-up	46.63 (34.46)	44.79 (35.59)	1.66 (2.22)	.45
12-wk follow-up	37.49 (36.60)	34.45 (35.97)	2.33 (2.26)	.30
** 20-wk follow-up**	**43.58 (38.23)**	**35.73 (36.66)**	**6.73 (2.34)**	**.004**
34-wk follow-up	41.01 (37.38)	38.44 (36.39)	1.98 (2.38)	.40
** Assist: provide information**				
8-wk follow-up	52.91 (34.39)	51.83 (35.94)	1.44 (2.24)	.52
12-wk follow-up	42.58 (38.47)	39.21 (38.59)	3.03 (2.28)	.18
** 20-wk follow-up**	47.99 (38.23)	39.35 (38.05)	**7.87 (2.36)**	**<.001**
34-wk follow-up	46.62 (38.54)	43.89 (38.47)	2.49 (2.40)	.30
** Assist: recommend medication**				
8-wk follow-up	39.80 (33.99)	39.66 (34.83)	-0.39 (2.08)	.85
12-wk follow-up	31.77 (34.16)	29.02 (33.92)	1.47 (2.11)	.48
** 20-wk follow-up**	37.50 (36.24)	27.89 (32.91)	**7.96 (2.19)**	**<.001**
34-wk follow-up	35.41 (35.30)	31.50 (33.22)	2.55 (2.23)	.25
** Arrange**				
8-wk follow-up	37.53 (34.32)	38.39 (35.20)	–1.03 (2.10)	.62
12-wk follow-up	28.89 (33.74)	30.10 (36.26)	–1.93 (2.13)	.36
** 20-wk follow-up**	**35.71 (36.17)**	**30.26 (35.59)**	**4.55 (2.21)**	**.040**
34-wk follow-up	32.98 (34.38)	31.99 (35.39)	0.48 (2.25)	.83
** Knowledge post-training**	634.58 (128.54)	648.54 (119.45)	13.98 (8.27)	.09

Bold suggested statistical significance.

^a^Mixed-effects statistical model, adjusting for age, working duration, gender, nationality, education level, hospital level, hospital nature, working department, professional title, and smoking status.

Secondary outcomes, encompassing self-reported continuous abstinence and smoking cessation initiation rates, showed no significant differences between groups at any follow-up point.

The study also examined the usage of the standard 5A’s intervention and knowledge related to smoking cessation. Both groups exhibited a notable increase in the utilization of various 5A components (“Assess,” “Assist: set a quit date,” “Assist: recommend cessation program,” “Assist: provide information,” “Assist: recommend medication,” and “Arrange”) at the 8-week follow-up compared to baseline. The intervention group demonstrated significantly higher utilization rates for all-5A’s components, except for “Assist: set a quit date,” at the 20-week follow-up. Significant differences in “Ask” and “Advise” were also noted at 8 and 12-week follow-ups, respectively.

### Explanatory Analysis

#### Factors Associated with Quitting From “WeChat WeQuit” Training in the Main Study

Table 3 compared the baseline characteristics of participants who completed(*n* = 509) and did not complete (*n* = 433) the “Wechat WeQuit” training. Those who dropped out from the training were more likely to be male and ex-smokers or current smokers compared to those who received the entire training.

**Table 3:** Baseline Characteristics of the Participants Who Received the Training or Not

Variable	Participants who did not receive entire training, *N* = 433^a^	Participants who received entire training, *N* = 509^a^	*p*-Value^b^
**Gender**			.043
Male	195 (45%)	196 (39%)	
Female	238 (55%)	313 (61%)	
**Age, y**	36.88 (8.15)	37.17 (8.11)	.44
**Nationality**			.80
Han	405 (94%)	474 (93%)	
Non-Han	28 (6.5%)	35 (6.9%)	
**Education level**			.050
Bachelor’s degree or below	330 (76%)	359 (71%)	
Masteral or doctoral degree	103 (24%)	150 (29%)	
**Working duration, y**	13.29 (9.14)	13.67 (8.98)	.37
**Hospital level**			.66
Tertiary hospital	260 (60%)	309 (61%)	
Secondary hospital	120 (28%)	130 (26%)	
Primary hospital	53 (12%)	70 (14%)	
**Hospital nature**			.44
Public hospital	397 (92%)	457 (90%)	
Private hospital	32 (7.4%)	43 (8.4%)	
Clinic	4 (0.9%)	9 (1.8%)	
**Major**			.10
Psychiatry or mental health	171 (39%)	228 (45%)	
Respiratory medicine	56 (13%)	74 (15%)	
Others	206 (48%)	207 (41%)	
**Professional title**			.10
Junior	161 (37%)	163 (32%)	
Middle	178 (41%)	208 (41%)	
Senior	94 (22%)	138 (27%)	
**Smoking status**			<.001
Nonsmoker	321 (74%)	444 (87%)	
Ex-smoker	87 (20%)	43 (8%)	
Current smoker	25 (6%)	22 (5%)	
**Utilization rate of standard 5A’s intervention,%**			
Ask	62.04 (37.15)	61.75 (38.86)	.90
Advice	64.19 (35.39)	64.70 (35.69)	.83
Assess	43.42 (33.04)	40.47 (34.16)	.18
Assist: set a quit date	29.76 (32.65)	27.04 (32.97)	.21
Assist: recommend cessation program	33.30 (34.38)	32.55 (34.54)	.74
Assist: provide information	38.63 (34.40)	36.54 (35.07)	.36
Assist: recommend medication	30.39 (32.21)	28.84 (33.13)	.47
Arrange	29.39 (32.31)	27.63(33.65)	.42

^a^ *n* (%); mean (SD).

^b^Pearson’s chi-squared test; Welch two sample *t* test.

#### Per-Protocol Analysis of the Main Study

In the per-protocol (PP) analysis, we included data from participants who completed the training, comprising 519 individuals in the control group and 509 in the intervention group. The outcomes of this analysis are detailed in Table 4. Consistent with the ITT analysis, the PP analysis revealed a statistically significant higher overall utilization rate of smoking cessation interventions in the intervention group (36.66%) compared to the control group (31.03%) at the 20-week follow-up (*p* = .003). Furthermore, the intervention group exhibited enhanced utilization rates for the components of the 5A’s intervention model, including “Ask,” “Advise,” “Assess,” “Assist: recommend cessation program,” “Assist: provide information,” and “Assist: recommend medication” at the 20-week follow-up. Additionally, the self-reported initiation rate of smoking cessation among patients treated by HSPs was also higher in the intervention group at the 20-week follow-up.

**Table 4.** Per-Protocol Analysis of Outcomes for All-Participants in the WeChat Intervention and Control Groups (*n* = 1028)

Outcome	WeChat intervention group, *n* = 509	Control group, *n* = 519	Adjusted mean difference (standard error)	*p*-value^a^
***Primary outcome***				
**Overall utilization rate, %**				
8-wk follow-up	41.13 (33.63)	42.64 (34.28)	–0.40 (2.20)	.85
12-wk follow-up	32.15 (33.61)	30.88 (34.85)	3.07 (2.22)	.17
** 20-wk follow-up**	**36.66 (34.78)**	**31.03 (34.56)**	**6.72 (2.29)**	**.003**
34-wk follow-up	35.51 (34.04)	33.84 (32.97)	2.24 (2.26)	.15
*** Secondary outcome***				
** Self-reported continuous abstinence rate of patients treated by HSPs, %**				
8-wk follow-up	4.38 (8.42)	5.28 (11.07)	–0.22 (1.58)	.88
12-wk follow-up	21.25 (28.05)	21.30 (29.09)	1.00 (1.79)	.58
20-wk follow-up	21.61 (28.18)	19.23 (26.49)	3.01 (1.84)	.10
34-wk follow-up	19.15 (27.36)	18.56 (26.58)	1.84 (1.87)	.32
** Self-reported initiation rates of smoking cessation among patients treated by HSPs, %**				
8-wk follow-up	31.46 (28.86)	33.70 (30.23)	–1.06 (1.92)	.58
12-wk follow-up	24.70 (28.74)	24.56 (30.49)	2.88 (1.96)	.14
** 20-wk follow-up**	**28.56 (29.87)**	**25.68 (29.97)**	**4.78 (2.01)**	**.018**
34-wk follow-up	28.29 (30.08)	27.41 (29.27)	3.62 (2.05)	.08
*** Other outcomes***				
** Utilization rate of 5A’s intervention, %**				
** Ask**				
8-wk follow-up	64.69 (34.69)	64.38 (36.25)	3.81 (2.67)	.15
** 12-wk follow-up**	51.61 (40.45)	48.01 (42.54)	**7.04 (2.72)**	**.010**
** 20-wk follow-up**	57.22 (39.70)	50.40 (42.22)	**10.23 (2.80)**	**<.001**
34-wk follow-up	53.63 (41.03)	53.40 (42.46)	3.84 (2.84)	.17
** Advice**				
8-wk follow-up	68.62 (34.71)	65.42 (37.33)	3.57 (2.70)	.19
** 12-wk follow-up**	54.14 (41.61)	48.88 (42.98)	**5.90 (2.75)**	**.032**
** 20-wk follow-up**	58.85 (40.96)	51.87 (43.20)	**7.70 (2.84)**	**.007**
34-wk follow-up	56.93 (41.86)	56.44 (42.62)	1.74 (2.89)	.54
** Assess**				
8-wk follow-up	52.00 (31.82)	49.88 (34.33)	0.64 (2.41)	.79
12-wk follow-up	41.31 (35.95)	38.06 (36.74)	2.25 (2.45)	.36
** 20-wk follow-up**	48.52 (37.63)	39.62 (37.62)	**7.21 (2.53)**	**.004**
34-wk follow-up	45.16 (37.27)	42.13 (37.04)	2.21 (2.56)	.39
** Assist: set a quit date**				
8-wk follow-up	38.02 (33.06)	38.47 (34.48)	-0.03 (2.28)	.16
12-wk follow-up	31.14 (33.91)	28.57 (33.74)	1.62 (2.10)	.44
20-wk follow-up	34.89 (35.08)	29.92 (33.47)	2.78 (2.18)	.20
34-wk follow-up	33.29 (34.26)	32.01 (33.22)	0.58 (2.21)	.79
** Assist: recommend cessation program**				
8-wk follow-up	46.23 (34.58)	44.49 (35.30)	-0.54 (2.45)	.82
12-wk follow-up	37.78 (36.46)	34.71 (36.21)	0.80 (2.50)	.75
** 20-wk follow-up**	43.79 (37.90)	35.95 (36.77)	**5.15 (2.58)**	**.046**
34-wk follow-up	41.30 (37.39)	38.24 (36.05)	0.88 (2.60)	.74
** Assist: provide information**				
8-wk follow-up	52.55 (34.69)	51.82 (35.70)	–0.13 (2.49)	.96
12-wk follow-up	42.50 (38.12)	39.01 (38.45)	2.29 (2.54)	.37
** 20-wk follow-up**	48.48 (37.86)	39.48 (38.33)	**7.42 (2.61)**	**.004**
34-wk follow-up	46.42 (38.31)	44.06 (38.26)	1.27 (2.64)	.63
** Assist: recommend medication**				
8-wk follow-up	39.81 (34.30)	39.74 (34.64)	–2.46 (2.31)	.29
12-wk follow-up	32.06 (34.23)	28.81 (33.89)	0.52 (2.36)	.83
** 20-wk follow-up**	38.18 (36.39)	28.20 (33.39)	**6.82 (2.43)**	**.005**
34-wk follow-up	35.46 (35.35)	31.52 (33.21)	1.30 (2.45)	.60
** Arrange**				
8-wk follow-up	36.68 (34.39)	38.11 (34.98)	–2.81 (2.31)	.22
12-wk follow-up	28.89 (33.73)	30.07 (36.11)	–2.72 (2.36)	.25
20-wk follow-up	35.87 (36.03)	29.75 (35.28)	4.47 (2.43)	.07
34-wk follow-up	32.74 (34.26)	32.14 (35.54)	–0.55 (2.46)	.82
** Knowledge post-training**	653.15 (115.86)	635.01 (128.38)	135.20 (8.48)	.07

Bold suggested statistical significance.

^a^Mixed-effects statistical model, adjusting for age, working duration, gender, nationality, education level, hospital level, hospital nature, working department, professional title, and smoking status.

## Discussion

Since its debut in 2011, WeChat has become China’s leading social media platform. It offers a more comprehensive and intuitive interaction than traditional text messaging, making it a promising channel for smoking cessation interventions. Our recent study suggested that WeChat-based smoking cessation intervention could effectively improve the cessation rate among Chinese smokers.^28^ In the present study, we evaluated the efficacy of a WeChat-based online training program, “WeChat WeQuit,” in enhancing the utilization of smoking cessation strategies among Chinese HSPs. HSPs who received the “WeChat WeQuit” training program outperformed the control group in the overall utilization rate of smoking cessation. The program also led to notable enhancements in the application-specific 5A’s framework components. Taken together, our study suggested that the “WeChat WeQuit” program was effective in improving the utilization rate of smoking cessations among Chinese HSPs.

Although participants generally held positive attitudes towards the program, nearly half of the participants did not complete the WeChat-based smoking cessation training, a drop out rate significantly higher than that observed in similar studies among American HSPs.^29,30^ These programs often included continuing medical education credits as incentives,^29–31^ which could motivate HSPs to complete the training. The drop out rate was also markedly higher than our recent research involving a WeChat-based intervention among Chinese smokers, which reported a drop out rate of around 15%.^28^ A possible explanation for this discrepancy could be the demanding schedules of Chinese HSPs, which may hinder their ability to complete the program. In the explanatory analysis, we found males and smokers were more likely to drop out of the training. Further qualitative studies, such as interviews or focus groups, were warranted to understand these specific barriers to training completion among Chinese HSPs.

The overall utilization rate of smoking cessation was low (approximately 30%–40%), which suggested that only two-fifths of the smokers treated by HSPs received smoking cessation intervention. This finding aligned with the 2018 China Adult Tobacco Survey Report (GATS), which found that 46.4% of smokers received cessation advice from Chinese HSPs.^32^ Notably, the GATS reports indicate a downward trend, with the proportion of smokers receiving cessation advice declining from 58.2% in 2015 to 46.4% in 2018. Similarly, a retrospective study of 4269 Chinese hypertensive patients demonstrated a decreasing incidence of physician-provided smoking cessation advice from 2015 to 2018.^33^ These findings highlighted the strong need to further enhance the utilization of smoking cessation strategies among Chinese HSPs.

Consistent with prior studies,^34,35^ “Ask” and “Advice” were the most frequently used 5A intervention strategies in Chinese HSPs. In contrast, the utilization rate of other 5A’ s components were relatively low. Studies in other cultural backgrounds have produced consistent results, finding that the utilization rate of “Assist” and “Arrange” was lower compared to “Ask,” “Advice,” and “Assess.”^36–38^

Recent studies suggested that digital education is at least as effective as traditional training in improving HSPs’ knowledge and skills for smoking cessation therapy.^12^ Our study further corroborated these insights, suggesting the “WeChat WeQuit” program could improve the knowledge of smoking cessation and utilization rates of “Assist,” “Arrange,” and “Assess.” The utilization rate of these 5A’s components was superior in the “WeChat WeQuit” group compared to the control group at the 20-week follow-up. Sarna et al. reported consistent results with us, finding that web-based educational programs could improve the employment of “Assess,” “Assist,” and “Arrange” in Chinese nurses.^39^ These results emphasized the potential of digital platforms in enhancing the delivery and effectiveness of smoking cessation interventions.

Notably, at the 34-week follow-up, there was no significant difference in 5A’s employment rates between the two groups. The lack of sustained differences at the 34-week follow-up could be indicative of challenges in maintaining long-term adherence to the practices learned through digital training. It highlights the need for continuous engagement and reinforcement strategies to ensure the enduring effectiveness of smoking cessation interventions. Future research could explore the factors contributing to this observed decline in utilization rates over time and investigate methods to sustain the impact of smoking cessation training among HSPs.

Several limitations should be acknowledged. First, the sample was mainly composed of females, nonsmokers, and HSPs working in psychiatry and mental health departments. Therefore, the generality of our findings required further studies to confirm. Second, there was a high drop out rate in both groups. However, both ITT and PP analysis demonstrated consistent results, suggesting the robustness of our findings. Explanatory analysis suggested that male gender and the smoking experience were associated with a higher drop out rate. Further studies are warranted to improve the acceptability of WeChat-based smoking cessation training in these populations. Third, all-outcomes were self-reported, leading to potential memory and social desirability bias.

In conclusion, our study suggested that the WeChat-based online training program “WeChat WeQuit” could be effective in improving the utilization rate of smoking cessation intervention among Chinese HSPs.

### Ethical Issues

The protocol was approved by the Ethics Committee of the Second Xiangya Hospital of Central South University (2018, No. S07) and the Ethics Committee of Sir Run Run Shaw Hospital, an affiliate of Zhejiang University, Medical College (NO. 20200508-33) and the studies were carried out in accordance with the Declaration of Helsinki. At the time of the initial screening interview, the study participants were required to read the informed consent (confirmation of an electronic version of written informed consent will be performed) and ask any questions they may have about it. At the same time, a researcher read the consent statement to every HSP during the recruitment process. Each participant received an explanation about the aims, importance, procedures, measurements, potential risks, and benefits of the study before recruitment. At the time of the initial screening interview and again during the orientation session preceding the study, participants were required to know about the consent form and may ask any questions they may have about it. Participation was completely voluntary. They could withdraw from the study at any time. Only the investigators can identify the personal information of participants and have access to the final trial dataset. In this single-blind, randomized trial, participants had a 50% chance to be selected to either a control group or a group that receives the “WeChat WeQuit” program. Participants in the control group could join in the “WeChat WeQuit” program after the study. Participants were given contact details of the study coordinator for their future questions and concerns. Thus, all-participants were able to contact the study team for extra advice or suggestions.

## Supplementary material

Supplementary material is available at *Nicotine and Tobacco Research* online.

ntae166_suppl_Supplementary_Data

## Data Availability

The data, anonymized study data, digital materials, code, and the laboratory log of this study can be found at https://osf.io/4h35q/.
