# Supplementary material for: Boosting Smoking Cessation Intervention Utilization in Chinese Health Care Providers: A Randomized Controlled Trial of the “WeChat WeQuit” Medical Education Program
Source: Nicotine Tob Res. 2024 Jul 31;27(1):61–72. doi: 10.1093/ntr/ntae166 (PMC11663801; doi:10.1093/ntr/ntae166)
Supplement: ntae166_suppl_Supplementary_Data [file ntae166_suppl_supplementary_data.zip › Table S3.docx]

Table S3. The liking, understanding, and utilization rate of smoking cessation knowledge before and post training in the intervention group (n=78)

| Knowledge | Pre-training^1^ | Post-training^1^ | Mean difference | p-value^2^ |
| --- | --- | --- | --- | --- |
| ***Week 1*** |  |  |  |  |
| **Smoking demographics** |  |  |  |  |
| Liking | 47.09 (29.67) | 70.58 (30.29) | 23.49 | <0.001 |
| Understanding | 37.01 (24.92) | 78.96 (17.06) | 41.95 | <0.001 |
| Utilization rate | 32.38 (24.21) | 68.3 (23.09) | 35.92 | <0.001 |
| **Health risks of smoking** |  |  |  |  |
| Liking | 52.1 (26.54) | 79.1 (20.38) | 27.00 | <0.001 |
| Understanding | 44.57 (25.74) | 79.09 (17.78) | 34.52 | <0.001 |
| Utilization rate | 36.3 (21.01) | 72.76 (20.85) | 36.46 | <0.001 |
| ***Week 2*** |  |  |  |  |
| **Nicotine dependence** |  |  |  |  |
| Liking | 42.33 (25.7) | 71.85 (23.65) | 29.52 | <0.001 |
| Understanding | 41.73 (25.16) | 77.27 (17.49) | 35.53 | <0.001 |
| Utilization rate | 38.62 (26.22) | 71.51 (24.6) | 32.89 | <0.001 |
| **The nature course of smoking** |  |  |  |  |
| Liking | 40.89 (28.62) | 71.7 (23.92) | 30.81 | <0.001 |
| Understanding | 39.49 (23.87) | 73.84 (19.4) | 34.34 | <0.001 |
| Utilization rate | 36.72 (25.61) | 69.96 (23.78) | 33.24 | <0.001 |
| **The criteria of nicotine dependence** |  |  |  |  |
| Liking | 39.04 (25.1) | 72.06 (22.31) | 33.03 | <0.001 |
| Understanding | 37.76 (23.65) | 76.32 (16.96) | 38.56 | <0.001 |
| Utilization rate | 38.51 (26.65) | 69.82 (25.36) | 31.32 | <0.001 |
| ***Week 3*** |  |  |  |  |
| **5A's and 5R's intervention** |  |  |  |  |
| Liking | 35.13 (26.6) | 74.62 (21.03) | 39.49 | <0.001 |
| Understanding | 32.78 (24.11) | 73.56 (20.21) | 40.77 | <0.001 |
| Utilization rate | 30.97 (24.63) | 69.54 (24.48) | 38.57 | <0.001 |
| **ABCs intervention** |  |  |  |  |
| Liking | 33.73 (25.83) | 71.27 (20.69) | 37.53 | <0.001 |
| Understanding | 28.57 (21.93) | 73.73 (19.53) | 45.16 | <0.001 |
| Utilization rate | 29.39 (23.35) | 69.08 (25.35) | 39.68 | <0.001 |
| **SBIRT intervention** |  |  |  |  |
| Liking | 33.44 (22.15) | 71.75 (21.34) | 38.30 | <0.001 |
| Understanding | 32.08 (23.5) | 71.7 (18.72) | 39.62 | <0.001 |
| Utilization rate | 32.11 (23.45) | 69.43 (24.64) | 37.32 | <0.001 |
| ***Week 4*** |  |  |  |  |
| **Increasing motivation** |  |  |  |  |
| Liking | 43.96 (26.57) | 77.71 (19.24) | 33.75 | <0.001 |
| Understanding | 37.71 (22.99) | 75.84 (19.5) | 38.13 | <0.001 |
| Utilization rate | 35.46 (22.86) | 76.13 (23.05) | 40.67 | <0.001 |
| **Decreasing triggers** |  |  |  |  |
| Liking | 38.57 (24.02) | 74.71 (20.32) | 36.14 | <0.001 |
| Understanding | 36.49 (23.14) | 76.94 (18.54) | 40.44 | <0.001 |
| Utilization rate | 35.67 (23.12) | 71.51 (24.06) | 35.84 | <0.001 |
| **Set a quit data** |  |  |  |  |
| Liking | 36.57 (25.68) | 73.99 (21.11) | 37.42 | <0.001 |
| Understanding | 34.56 (23.38) | 73.68 (19.98) | 39.13 | <0.001 |
| Utilization rate | 36.84 (26.81) | 69.81 (25.4) | 32.97 | <0.001 |
| **Not ready to quit right now** |  |  |  |  |
| Liking | 37.63 (26.49) | 74.05 (22.28) | 36.42 | <0.001 |
| Understanding | 34.38 (23.78) | 75.41 (22.12) | 41.03 | <0.001 |
| Utilization rate | 34.27 (24.68) | 71.58 (24.4) | 37.32 | <0.001 |
| **Relax training** |  |  |  |  |
| Liking | 47.2 (28.29) | 78.41 (19.3) | 31.20 | <0.001 |
| Understanding | 43.59 (27.08) | 79.23 (19.7) | 35.63 | <0.001 |
| Utilization rate | 43.15 (27.99) | 76.22 (22.56) | 33.06 | <0.001 |
| ***Week 5*** |  |  |  |  |
| **Individual counselling and psychotherapy** |  |  |  |  |
| Liking | 42.73 (26.68) | 76.9 (20.42) | 34.16 | <0.001 |
| Understanding | 38.81 (25.22) | 73.58 (19.22) | 34.77 | <0.001 |
| Utilization rate | 38.71 (26.8) | 71.28 (24.99) | 32.57 | <0.001 |
| **Test-message based smoking quit** |  |  |  |  |
| Liking | 39.01 (26.78) | 70.65 (22.83) | 31.63 | <0.001 |
| Understanding | 35.43 (24.6) | 71.53 (22.43) | 36.10 | <0.001 |
| Utilization rate | 33.09 (26.72) | 64.38 (27.52) | 31.29 | <0.001 |
| **Group counselling and psychotherapy** |  |  |  |  |
| Liking | 37.71 (25.9) | 71.04 (23.6) | 33.33 | <0.001 |
| Understanding | 34.08 (24.87) | 69.22 (23.35) | 35.14 | <0.001 |
| Utilization rate | 32.15 (25.13) | 62.38 (29.33) | 30.23 | <0.001 |
| **Mindfulness training** |  |  |  |  |
| Liking | 39.91 (25.44) | 72.89 (22.06) | 32.97 | <0.001 |
| Understanding | 35.68 (24.49) | 69.89 (22.97) | 34.20 | <0.001 |
| Utilization rate | 36.54 (27.45) | 64.44 (27.5) | 27.90 | <0.001 |
| ***Week 6*** |  |  |  |  |
| **Nicotine replacement therapy** |  |  |  |  |
| Liking | 40.38 (27.4) | 70.85 (23.55) | 30.47 | <0.001 |
| Understanding | 37.8 (24.85) | 75.23 (19.82) | 37.43 | <0.001 |
| Utilization rate | 33.2 (24.41) | 63.09 (28.29) | 29.89 | <0.001 |
| **Non-nicotine replacement therapy** |  |  |  |  |
| Liking | 38.92 (26.88) | 71.37 (23.6) | 32.44 | <0.001 |
| Understanding | 35.28 (25.4) | 68.92 (23.81) | 33.65 | <0.001 |
| Utilization rate | 35.37 (27.14) | 64.78 (27.93) | 29.42 | <0.001 |
| **E-cigarette for quitting smoking** |  |  |  |  |
| Liking | 38.25 (26.89) | 65.0 (27.61) | 26.75 | <0.001 |
| Understanding | 35.9 (23.59) | 69.23 (22.65) | 33.33 | <0.001 |
| Utilization rate | 35.09 (26.46) | 62.09 (29.61) | 27.00 | <0.001 |
| **The efficacy of smoking cessation** |  |  |  |  |
| Liking | 38.59 (25.59) | 73.37 (21.28) | 34.77 | <0.001 |
| Understanding | 35.08 (24.78) | 72.22 (20.07) | 37.14 | <0.001 |
| Utilization rate | 35.47 (25.53) | 68.9 (26.19) | 33.43 | <0.001 |
| ***Week 7*** |  |  |  |  |
| **Smoking cessation for adolescents** |  |  |  |  |
| Liking | 44.52 (30.36) | 75.61 (23.05) | 31.09 | <0.001 |
| Understanding | 37.44 (24.96) | 69.82 (23.34) | 32.38 | <0.001 |
| Utilization rate | 34.72 (24.26) | 64.2 (24.96) | 29.48 | <0.001 |
| **Smoking cessation for female** |  |  |  |  |
| Liking | 38.99 (26.18) | 68.9 (25.17) | 29.91 | <0.001 |
| Understanding | 32.9 (24.28) | 68.3 (23.1) | 35.41 | <0.001 |
| Utilization rate | 33.1 (23.98) | 62.57 (27.25) | 29.47 | <0.001 |
| **Smoking cessation for pregnancy** |  |  |  |  |
| Liking | 36.05 (25.59) | 71.66 (24.34) | 35.61 | <0.001 |
| Understanding | 32.1 (21.96) | 67.49 (24.6) | 35.39 | <0.001 |
| Utilization rate | 33.48 (24.64) | 64.73 (25.87) | 31.25 | <0.001 |
| **Smoking cessation for postpartum** |  |  |  |  |
| Liking | 37.63 (28.6) | 66.75 (25.93) | 29.11 | <0.001 |
| Understanding | 31.92 (22.98) | 68.28 (23.29) | 36.35 | <0.001 |
| Utilization rate | 29.92 (23.51) | 63.24 (28.09) | 33.32 | <0.001 |
| **Smoking cessation for patients with physical disorder** |  |  |  |  |
| Liking | 40.38 (27.03) | 73.03 (22.78) | 32.65 | <0.001 |
| Understanding | 32.95 (21.88) | 71.82 (20.57) | 38.87 | <0.001 |
| Utilization rate | 34.61 (23.27) | 66.44 (25.41) | 31.84 | <0.001 |
| **Smoking cessation for patients with mental disorder** |  |  |  |  |
| Liking | 38.29 (26.81) | 72.04 (23.56) | 33.75 | <0.001 |
| Understanding | 34.52 (22.09) | 71.66 (22.21) | 37.14 | <0.001 |
| Utilization rate | 34.24 (24.12) | 66.56 (27.04) | 32.32 | <0.001 |
| **The interaction of smoking and medication** |  |  |  |  |
| Liking | 37.2 (24.91) | 71.29 (25.02) | 34.09 | <0.001 |
| Understanding | 33.97 (23.03) | 70.35 (22.81) | 36.38 | <0.001 |
| Utilization rate | 33.24 (23.73) | 64.82 (26.46) | 31.58 | <0.001 |
| ***Week 8*** |  |  |  |  |
| **Dealing with withdrawal symptoms** |  |  |  |  |
| Liking | 40.24 (27.45) | 73.87 (21.18) | 33.63 | <0.001 |
| Understanding | 36.91 (22.94) | 73.67 (19.57) | 36.76 | <0.001 |
| Utilization rate | 36.24 (23.35) | 68.57 (24.81) | 32.33 | <0.001 |
| **Addressing weight gain concerns** |  |  |  |  |
| Liking | 43.19 (25.85) | 70.68 (21.81) | 27.49 | <0.001 |
| Understanding | 37.27 (23.86) | 71.43 (19.54) | 34.16 | <0.001 |
| Utilization rate | 33.8 (22.86) | 68.47 (25.55) | 34.67 | <0.001 |
| **Dealing with lapses** |  |  |  |  |
| Liking | 43.58 (29.5) | 73.92 (25.24) | 30.34 | <0.001 |
| Understanding | 35.13 (24.57) | 73.0 (20.66) | 37.87 | <0.001 |
| Utilization rate | 34.96 (25.04) | 66.7 (26.59) | 31.73 | <0.001 |
| **Maintaining long-term abstinence** |  |  |  |  |
| Liking | 40.65 (26.99) | 71.34 (22.43) | 30.70 | <0.001 |
| Understanding | 36.78 (26.14) | 71.66 (21.9) | 34.87 | <0.001 |
| Utilization rate | 34.11 (24.06) | 66.48 (26.84) | 32.37 | <0.001 |

|  |
| --- |
|  |
| 1 Mean (SD)  2 Pair t-tests |
|  |
