# Supplementary material for: Boosting Smoking Cessation Intervention Utilization in Chinese Health Care Providers: A Randomized Controlled Trial of the “WeChat WeQuit” Medical Education Program
Source: Nicotine Tob Res. 2024 Jul 31;27(1):61–72. doi: 10.1093/ntr/ntae166 (PMC11663801; doi:10.1093/ntr/ntae166)
Supplement: ntae166_suppl_Supplementary_Data [file ntae166_suppl_supplementary_data.zip › Table S4.docx]

Table S4. The efficacy of “Wechat Wequit” Intervention in improving the utilization rate of 5A’s intervention

| 5A’s intervention | Mean change in the intervention group^1^ | Mean change in the control group^1^ | Mean difference | p-value^2^ |
| --- | --- | --- | --- | --- |
| **Ask** | -3.01 (38.02) | -2.84 (37.52) | 0.93 | 0.98 |
| **Advice** | -0.84 (40.44) | -1.63 (42.17) | 0.79 | 0.91 |
| **Assess** | 17.90 (36.43) | 14.43 (44.81) | 3.47 | 0.61 |
| **Assist: set a quit data** | 33.83 (35.68) | 21.72 (44.80) | 12.11 | 0.07 |
| **Assist: recommend cessation program** | 31.83 (35.23) | 13.90 (42.06) | 17.93 | 0.006 |
| **Assist: provide information** | 24.03 (34.80) | 9.84 (38.53) | 14.19 | 0.020 |
| **Assist: recommend medication** | 32.00 (36.72) | 16.51 (39.27) | 15.49 | 0.014 |
| **Arrange** | 35.33 (35.00) | 21.79 (38.71) | 13.54 | 0.027 |

| 1 Mean (SD)  2 Student t-tests |
| --- |
