# Supplementary material for: Boosting Smoking Cessation Intervention Utilization in Chinese Health Care Providers: A Randomized Controlled Trial of the “WeChat WeQuit” Medical Education Program
Source: Nicotine Tob Res. 2024 Jul 31;27(1):61–72. doi: 10.1093/ntr/ntae166 (PMC11663801; doi:10.1093/ntr/ntae166)
Supplement: ntae166_suppl_Supplementary_Data [file ntae166_suppl_supplementary_data.zip › Appendix.docx]

**Appendix 1**

**The 5 A’S of intervention**

1. **ASK - 1 minute**

Ask patient to describe their smoking status.

A. I NEVER smoked or smoked LESS THAN 100 cigarettes.

B. I stopped smoking more than 2 weeks ago but less than 1 year ago.

C. I stopped smoking more than 1 year ago.

D. I smoke regularly/not thinking of quitting in the next 30 days.

If B or C, reinforce their decision to quit, congratulate and encourage.

If D, document smoking status on their chart. Begin steps below.

1. **ADVISE - 1 minute**

Provide clear, strong advice to quit with personalized messages about the impact of smoking on health; urge every tobacco user to quit.

1. **ASSESS - 1 minute**

Assess the willingness to make a quit attempt within 30 days.

- Patient is willing to make a quit attempt in the next 14-30 days
- Patient is not willing to make a quit attempt (review the 5 R’s)

1. **ASSIST - 3 minutes**

Recommend the use of approved pharmacotherapy.

Refer to currently available cessation services.

**AND/OR**

Help the patient develop a quit plan.

Provide problem-solving methods and skills for cessation.

Provide social support as a part of the treatment.

Help patient obtain extra treatment/social support for quitting in the smoker’s environment.

Recommend the use of approved pharmacotherapy.

Provide self-help smoking cessation materials.

Provide relapse prevention.

1. **ARRANGE - 1 minute +**

Assess smoking status every visit, reinforce/encourage cessation.

**Appendix 2**

**The 5 R’S of motivation**

**Relevance - 1 minute**

Ask patient about how quitting may be personally relevant. Encourage the patient to indicate why quitting is personally relevant, being as specific as possible. Motivational information has the greatest impact if it is relevant to a patient’s disease status or risk, family or social situation (e.g., having children in the home), health concerns, age, gender, and other important patient characteristics (e.g., prior quitting experience, personal barriers to cessation). Examples of relevance include:

- Longer and better quality of life
- Extra money
- People you live with will be healthier
- Decrease chance of heart attack, stroke or cancer
- If pregnant, improves chance of healthy baby

**Risks - 1 minute**

Ask the patient about their perception of short-term, long-term and environmental risks of continued use. The HSPs may suggest and highlight those that seem most relevant to the patient. The HSPs should emphasize that smoking low-tar/low-nicotine cigarettes or use of other forms of tobacco (e.g., E-cigarettes, smokeless tobacco, cigars, and pipes) will not eliminate these risks. Examples of risks are:

- Acute risks: shortness of breath, exacerbation of asthma, increased risk of respiratory infections, harm to pregnancy, impotence, infertility.
- Long-term risks: heart attacks and strokes, lung and other cancers (e.g., larynx, oral cavity, pharynx, esophagus, pancreas, stomach, kidney, bladder, cervix and acute myelocytic leukemia), chronic obstructive pulmonary diseases (chronic bronchitis and emphysema), osteoporosis, long-term disability and need for extended care.
- Environmental risks: increased risk of lung cancer and heart disease in spouses; increased risk for low birth weight, sudden infant death syndrome (SIDS), asthma, middle ear disease, and respiratory infections in children of smokers.

**Rewards - 1 minute**

Ask the patient about perceived benefits/rewards for quitting tobacco use. The HSPs may suggest and highlight those that seem most relevant to the patient. Examples of rewards follow:

- Health (self & others)
- Food taste
- Sense of smell
- Feel better
- Example to others
- Additional years of life
- Saving money
- Performing better in physical activities
- Improved appearance including reduced wrinkling/aging of skin and whiter teeth

**Roadblocks - 3 minutes +**

The HSPs should ask the patient to identify barriers or impediments to quitting and provide treatment (problem-solving counseling, medication) that could address barriers. Typical barriers might include:

- Withdrawal symptoms
- Fear of failure
- Sleep problems
- Weight gain
- Lack of support
- Depression and anxiety
- Enjoyment of tobacco
- Limited knowledge of effective treatment options

**Repetition - 1 minute +**

Respectfully repeat the 5 R’s each visit, providing motivation and information. Tobacco users who have failed in previous quit attempts should be told that most people make repeated quit attempts before they are successful.

**Appendix 3**

**Questions for assessing program acceptability**

1. Overall rating of the program

A. Like very much

B. Like somewhat

C. Neutral

D. Dislike somewhat

E. Dislike very much

2. Appraisal of program--likelihood of applying program for smoking patients

A. Very likely

B. Somewhat likely

C. Neutral

D. Unlikely

E. Not at all likely

3. Appraisal of program--likelihood of recommending program to other HSPs

A. Very likely

B. Somewhat likely

C. Neutral

D. Unlikely

E. Not at all likely

4. I would not have been able to help patients quit without the program

A. Strongly agree

B. Agree

C. Neutral

D. Disagree

E. Strongly disagree

5. The program made it easier to help patients quit smoking

A. Strongly agree

B. Agree

C. Neutral

D. Disagree

E. Strongly disagree

6. The program disrupted my daily schedule

A. Strongly agree

B. Agree

C. Neutral

D. Disagree

E. Strongly disagree

7. Appraisal of WeChat-based messages

7.1 Frequency of reading messages

Will be reported on a 10-point scale ranging from 1 (never) to 10 (always).

|  |  |  |  |  |  |  |  |  |  |
| --- | --- | --- | --- | --- | --- | --- | --- | --- | --- |

0 1 2 3 4 5 6 7 8 9 10

7.2 I received too many messages

A. Strongly agree

B. Agree

C. Neutral

D. Disagree

E. Strongly disagree

8. The messages talked about what I was experiencing to learn

A. Strongly agree

B. Agree

C. Neutral

D. Disagree

E. Strongly disagree

**Appendix 4**

**Knowledge about behavioural and pharmacotherapy interventions for smoking cessation before and after 8 weeks will be on a 100-point scale**

**Week 1:** Smoking demographics and the health risks of smoking

**Week 2:** Nicotine dependence, the nature course of smoking, the criteria of nicotine dependence

**Week 3:** Intervention: 5A's and 5R's intervention, ABCs (Ask, Brief advice and Cessation support) intervention, SBIRT (Screening, brief intervention, and referral to treatment) intervention

**Week 4:** Quitting preparation: increasing motivation, decreasing triggers, set a quit data, not ready to quit right now, relaxation training

**Week 5:** Psychological and behavioural intervention: Individual counseling and psychotherapy, Group counseling and psychotherapy.

**Week 6:** Pharmacotherapy: nicotine replacement therapy and other medications (e.g. Bupropion and Varenicline)

**Week 7:** Cessation for subpopulation (e.g. women, adolescents, pregnancy and postpartum) and smokers with physical (e.g. cancers, cardiovascular diseases) or mental illness (e.g. schizophrenia, depression, anxiety, drug dependence)

**Week 8:** After quitting: dealing with withdrawal symptoms, addressing weight gain concerns, dealing with lapses, maintaining long-term abstinence

**Appendix 5**

**The utilization rate of interventions for smoking patients
 1. The utilization rate of the 5 A’S**

A.   Ask

B.   Advise
 C.   Assess: such as

- Willingness or motivation
- Preparation
- Self-efficacy

D.   Assist

- Set a quit data
- Recommend cessation program
- Provide cessation information
- Discuss or prescribe medications, such as first-line medications:
   – nicotine replacement therapy
   – Bupropion SR
   – Varenicline

E.    Arrange: such as

- Face to face visits
- Telephone follow-up
- Follow-up by WeChat, QQ, email et al.

**2. The overall utilization rate: smokers treated/ smokers seen**

**3. Smoking abstinence rate: the proportion of treated smoking patients with abstinence**
