# Supplementary material for: Boosting Smoking Cessation Intervention Utilization in Chinese Health Care Providers: A Randomized Controlled Trial of the “WeChat WeQuit” Medical Education Program
Source: Nicotine Tob Res. 2024 Jul 31;27(1):61–72. doi: 10.1093/ntr/ntae166 (PMC11663801; doi:10.1093/ntr/ntae166)
Supplement: ntae166_suppl_Supplementary_Data [file ntae166_suppl_supplementary_data.zip › Supplementary .docx]

**Supplementary materials for “Boosting Smoking Cessation Intervention Utilization in Chinese Healthcare Providers: A Randomized Controlled Trial of the 'WeChat WeQuit' Medical Education Program”**

**The methods of the pilot study**

The pilot study is to develop a 'WeChat WeQuit' program that aims at increasing HSPs’ utilization of behavioural and pharmacotherapy interventions for cigarette smoking cessation in China. The pilot study is to assess the content of both intervention group and control group messages that will be sent by WeChat in this study; quit message types and number of messages per quitting stage; how often messages will be received and read by HSPs; HSPs’ views on the intervention messages and their delivery; the proportion of HSPs and smokers who are contactable at follow-up and who provide outcome variables; sample size assessment for the main study; the knowledge about behavioural and pharmacotherapy interventions for smoking cessation before and after 8 weeks. The basis of the selection of questions on ‘knowledge’ was mainly based on clinical practice guideline for treating tobacco use and dependence24; and preliminary examination of the feasibility and effectiveness of the 'WeChat WeQuit' program in China. A total of 10% of the whole sample size (about 200 participants) were conducted during pilot study. Figure S1 displayed the flowchart of the pilot study. A total of approximately 200 HSPs were recruited during pilot study. Participants were selected to either a control group or to a group that receives a smoking cessation educational program. The pilot study held three main objectives, as detailed below:

**Develop the 'WeChat WeQuit' program:** Development of behavioural and pharmacotherapy interventions messages will be mainly based on clinical practice guidelines for treating Tobacco use and dependence^1^, as well as based on previous studies in other countries, and smokers and smoking cessation professionals in China. The 5 A’S of intervention are shown in **Appendix 1** and the 5 R’S of motivation are shown in **Appendix 2**. It took approximately one to two months to develop the program.

**Examine the feasibility and acceptability of 'WeChat WeQuit':** The feasibility and acceptability of the 'WeChat WeQuit' program was examined during the whole period of the pilot study stage. Questions for assessing program acceptability are shown in **Appendix 3**. Examinations will include test-retest reliability and internal consistency of the instruments; acceptability of the message contexts; quit message types and number of messages per quitting stage; how often messages will be received and read by HSPs and HSPs’ views on the intervention messages and their delivery; the proportion of HSPs and smokers who are contactable at follow-up and who provide outcome variables; sample size assessment for the main study; the knowledge about behavioural and pharmacotherapy interventions for smoking cessation before and after 8 weeks, which was mainly based on *clinical practice guideline for treating tobacco use and dependence*^1^; and preliminarily examination of the feasibility and effectiveness of the 'WeChat WeQuit' program in China. Knowledge about behavioural and pharmacotherapy interventions for smoking cessation before and after 8 weeks is shown in **Appendix 4**. The utilization rate of interventions for smoking patients is shown in **Appendix 5**.

**Assess the efficacy of 'WeChat WeQuit' intervention:** The pilot study was implemented with approximate 200 participants (100 HSPs in the intervention group and 100 HSPs in the control group), and feedback (such as acceptability of messages, context and number of messages/day) from participants were collected to improve the quality of the 'WeChat WeQuit' program. The pilot result of the utilization rate of interventions for smoking patients (see **Appendix 5**) and the assessment of HSPs’ liking and understanding of each week’s Knowledge about behavioural and pharmacotherapy interventions for smoking cessation.

**The results of the pilot study**

**Baseline characteristics of participants in the pilot study**

Two hundred and sixty-three individuals were recruited and screened in April 2019 (Figure 1). Among the participants, 235 HSPs met the inclusion criteria and were randomized into the Wechat Wequit intervention group (n=122) and control group (n=113). The baseline demographic information was presented in Table S1. No significant difference in demographic information was observed between the intervention and the control group. The sample was sex-balanced, with a slight majority being female (51%), and an average age of 36.45 years. The majority of participants were of Han ethnicity (92%) and held a Bachelor's degree or below (69%). Participants predominantly worked in tertiary care hospitals (55%) and were early to mid-career professionals, with the majority holding junior professional titles (77%). Most of the participants worked in the department of psychiatry and mental health (62%) and were non-smokers (73%). The utilization rate of the standard 5A’s intervention varied across the specific actions, with no statistically significant differences between the intervention and control groups.

**The completion rate, feasibility, and acceptability of the “Wechat Wequit” program**

The "Wechat Wequit" training program demonstrated a commendable completion rate, with nearly 70% (n=84) of participants completing at least 5 out of the 8 lessons. Among all the participants, 98 (82%) provided valid responses regarding their satisfaction with the intervention post-training, as outlined in Table S2. The intervention's feasibility and acceptability were substantiated by a high approval rating, with 68% (n=69) expressing strong liking ("Like very much") and 25% (n=24) indicating moderate satisfaction ("Like somewhat"). Minimal disruption to daily life was reported by 7% (n=7) of the HSPs, while a substantial majority (97%, n=95) would advocate for the program's recommendation to other HSPs.

The program also received positive reviews for its perceived efficacy; 94% (n=92) of participants felt they were "very likely" or "somewhat likely" to apply the strategies learned to assist smoking patients. Moreover, about 84% acknowledged their reliance on the program for acquiring effective smoking cessation counselling skills, asserting they would be at a loss without this training. Additionally, 95% testified that the program made it easier to help patients quit smoking.

Regarding content and frequency of “Wechat Wequit” messages, around 70% of participants read the "Wechat Wequit" messages very frequently (scoring 8 or above), and 96% (n=94) concurred that the message content aligned with their learning expectations. However, 30% of the participants reported that they received too many messages.

**Effectiveness of “Wechat Wequit” in the pilot study**

The pre-and post-training liking, understanding, and utilization rate of the smoking cessation training knowledge was assessed. Validated responses were received from 65% (n=77) of HSPs in the intervention group (Table S3), showing significant enhancements in the appreciation, comprehension, and application of the knowledge imparted throughout the 8-week program (all p<0.001).

70 HSPs in the intervention group and 81 HSPs in the control group provided data on the post-training utilization rate of 5A’s standard intervention (Table S4). Both groups demonstrated substantial improvements in the utilization rates of the 5A’s components: “Assess,” “Assist: set a quit date,” “Assist: recommend cessation program,” “Assist: provide information,” “Assist: recommend medication,” and “Arrange.” Noteworthy, the "Wechat Wequit" intervention group showed superior enhancements over the control group in the utilization rates of “Assist: recommend cessation program” (29.83 vs 14.36, p=0.01), “Assist: provide information” (22.97 vs 10.56, p=0.031), “Assist: recommend medication” (30.39 vs 17.67, p=0.026) and “Arrange.” (32.85 vs 21.22, p=0.036).

Reference

1. Tobacco TCPGT. A clinical practice guideline for treating tobacco use and dependence: 2008 update: a US public health service report. *American journal of preventive medicine.* 2008;35(2):158.
