# Supplementary material for: Boosting Smoking Cessation Intervention Utilization in Chinese Health Care Providers: A Randomized Controlled Trial of the “WeChat WeQuit” Medical Education Program
Source: Nicotine Tob Res. 2024 Jul 31;27(1):61–72. doi: 10.1093/ntr/ntae166 (PMC11663801; doi:10.1093/ntr/ntae166)
Supplement: ntae166_suppl_Supplementary_Data [file ntae166_suppl_supplementary_data.zip › Table S1 Pilot study sample characteristics.docx]

Table S1 Sample characteristics of the participants in the pilot study

| **Characteristic** | **Group** | | | **p-value^2^** |
| --- | --- | --- | --- | --- |
|  | **Overall, N = 235^1^** | **“Wechat Wequit” Intervention, N = 122^1^** | **Control, N = 113^1^** |  |
| **Gender** |  |  |  | 0.26 |
| Male | 115 (49%) | 64 (52%) | 51 (45%) |  |
| Female | 120 (51%) | 58 (48%) | 62 (55%) |  |
| **Age, year** | 36.45 (8.13) | 35.85 (8.09) | 37.10 (8.15) | 0.24 |
| **Nationality** |  |  |  | 0.51 |
| Han | 217 (92%) | 114 (93%) | 103 (91%) |  |
| Non-Han | 18 (7.7%) | 8 (6.6%) | 10 (8.8%) |  |
| **Education level** |  |  |  | 0.53 |
| Bachelor’s degree or below | 163(69%) | 87 (71%) | 76 (67%) |  |
| Masteral or doctoral degree | 72 (31%) | 35 (29%) | 37 (33%) |  |
| **Working duration, year** | 12.94 (8.93) | 12.26 (8.76) | 13.68 (9.09) | 0.23 |
| **Hospital level** |  |  |  | 0.64 |
| Tertiary hospital | 129 (55%) | 63 (52%) | 66 (58%) |  |
| Secondary hospital | 63 (27%) | 34 (28%) | 29 (26%) |  |
| Primary hospital | 43 (18%) | 25 (21%) | 18 (16%) |  |
| **Hospital nature** |  |  |  | 0.49 |
| Public hospital | 188 (80%) | 96 (79%) | 92 (81%) |  |
| Private hospital | 25 (11%) | 12 (10%) | 13 (12%) |  |
| Clinic | 22 (9%) | 14 (11%) | 8 (7%) |  |
| **Department** |  |  |  | 0.82 |
| Others | 82 (35%) | 44 (36%) | 38 (34%) |  |
| Psychiatry or mental health | 146 (62%) | 75 (61%) | 71 (63%) |  |
| Respiratory medicine | 7 (3.0%) | 3 (2.5%) | 4 (3.5%) |  |
| **Professional title** |  |  |  | 0.74 |
| Junior | 182 (77%) | 92 (75%) | 90 (80%) |  |
| Middle | 39 (17%) | 22 (18%) | 17 (15%) |  |
| Senior | 14 (6.0%) | 8 (6.6%) | 6 (5.3%) |  |
| **Smoking status** |  |  |  | 0.088 |
| Non-smoker | 171 (73%) | 82 (67%) | 89 (79%) |  |
| Ex-smoker | 52 (22%) | 31 (25%) | 21 (19%) |  |
| Current smoker | 12 (5.1%) | 9 (7.4%) | 3 (2.7%) |  |
| **Utilization rate of standard 5A’s intervention,%** |  |  |  |  |
| Ask | 65.12 (34.31) | 65.63 (35.16) | 64.57 (33.51) | 0.81 |
| Advice | 66.95 (33.08) | 67.70 (32.90) | 66.14 (33.40) | 0.72 |
| Assess | 47.89 (34.71) | 49.10 (35.55) | 46.58 (33.89) | 0.58 |
| Assist: set a quit data | 34.31 (36.10) | 33.40 (35.65) | 35.29 (36.71) | 0.69 |
| Assist: recommend cessation program | 41.85 (36.80) | 37.99 (35.13) | 46.02 (38.25) | 0.10 |
| Assist: provide information | 47.58 (36.60) | 44.63 (36.14) | 50.77 (36.99) | 0.20 |
| Assist: recommend medication | 37.52 (35.34) | 36.23 (35.27) | 38.91 (36.51) | 0.56 |
| Arrange | 34.20 (34.70) | 34.27 (35.34) | 34.12 (34.15) | 0.97 |
| ^1^n (%); Mean (SD) | | | | |
| ^2^Pearson's Chi-squared test; Welch Two Sample t-test; Fisher's exact test | | | | |
