# Supplementary material for: Boosting Smoking Cessation Intervention Utilization in Chinese Health Care Providers: A Randomized Controlled Trial of the “WeChat WeQuit” Medical Education Program
Source: Nicotine Tob Res. 2024 Jul 31;27(1):61–72. doi: 10.1093/ntr/ntae166 (PMC11663801; doi:10.1093/ntr/ntae166)
Supplement: ntae166_suppl_Supplementary_Data [file ntae166_suppl_supplementary_data.zip › Table S2 Satisfaction with the programme.docx]

Table S2 The feasibility and acceptability of “Wechat Wequit” program (n=98)

| Characteristics | Response |
| --- | --- |
| **Overall rating of the program** |  |
| Like very much | 68 (69%) |
| Like somewhat | 24 (25%) |
| Neutral | 4(4%) |
| Dislike somewhat | 2 (2%) |
| Dislike very much | 0 (0%) |
| **Appraisal of program--likelihood of applying program for smoking patients** |  |
| Very likely | 49 (50%) |
| Somewhat likely | 43 (44%) |
| Neutral | 4 (4%) |
| Unlikely | 2 (2%) |
| Not at all likely | 0 (0%) |
| **Appraisal of program--likelihood of recommending program to other HSPs** |  |
| Very likely | 46 (47%) |
| Somewhat likely | 49 (50%) |
| Neutral | 1 (1%) |
| Unlikely | 2 (2%) |
| Not at all likely | 0 (0%) |
| **I would not have been able to help patients quit without the program** |  |
| Strongly agree | 34 (32%) |
| Agree | 51 (52%) |
| Neutral | 10 (10%) |
| Disagree | 4 (4%) |
| Strongly disagree | 2 (2%) |
| **The program made it easier to help patients quit smoking** |  |
| Strongly agree | 55 (56%) |
| Agree | 38 (39%) |
| Neutral | 4 (4%) |
| Disagree | 1 (1%) |
| Strongly disagree | 0 (0%) |
| **The program disrupted my daily schedule** |  |
| Strongly agree | 4 (4%) |
| Agree | 3 (3%) |
| Neutral | 5 (5%) |
| Disagree | 63 (64%) |
| Strongly disagree | 23 (24%) |
| **Frequency of reading messages^1^** | 8.15 (1.79) |
| **I received too many messages** |  |
| Strongly agree | 8 (8%) |
| Agree | 21 (21%) |
| Neutral | 28 (29%) |
| Disagree | 31 (32%) |
| Strongly disagree | 10 (10%) |
| **The messages talked about what I was expecting to learn** |  |
| Strongly agree | 40 (41%) |
| Agree | 54 (55%) |
| Neutral | 3 (3%) |
| Disagree | 0 (0%) |
| Strongly disagree | 1 (1%) |

1 1=never, 10=always
