# Supplementary figures and images for: Boosting Smoking Cessation Intervention Utilization in Chinese Health Care Providers: A Randomized Controlled Trial of the “WeChat WeQuit” Medical Education Program
Source: Nicotine Tob Res. 2024 Jul 31;27(1):61–72. doi: 10.1093/ntr/ntae166 (PMC11663801; doi:10.1093/ntr/ntae166)

Figure S1 Flowdiagram for the pilot study


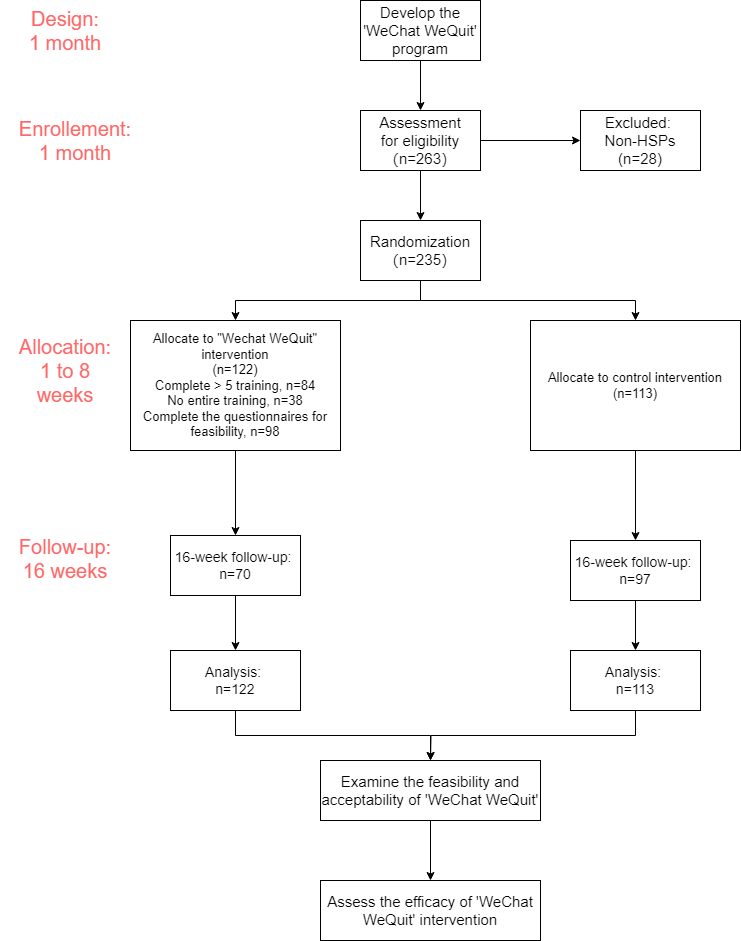

Supplement: ntae166_suppl_Supplementary_Data [file ntae166_suppl_supplementary_data.zip › Figure S1 Flowgram.docx]
